# Dream habits in a large cohort of preteens and their relation to sleep and nocturnal awakenings

**DOI:** 10.1111/jsr.14339

**Published:** 2024-09-11

**Authors:** Jean‐Baptiste Eichenlaub, Romain Bouet, Mathieu Pinelli, Sophie Portrat

**Affiliations:** ^1^ Univ. Grenoble Alpes, Univ. Savoie Mont Blanc, CNRS, LPNC Grenoble France; ^2^ Institut Universitaire de France (IUF) Paris France; ^3^ Lyon Neuroscience Research Center INSERM U1028/CNRS UMR 5292 Lyon France; ^4^ Univ. Grenoble Alpes, LaRAC Grenoble France

**Keywords:** dreaming, emotions, gender difference, nocturnal awakenings, preteens, sleep

## Abstract

The present study examined dream habits, and their relation to sleep patterns, in 1151 preteens (597 boys; 554 girls; 11.31 ± 0.62 years old). Dream questionnaires assessed the frequency of dream recall, nightmare, and lucid dream, as well as the intensity of emotions experienced in dreams. Sleep variables included sleep duration and efficiency, but also different measurements of nocturnal awakenings. Among the preteens, 49.21% of them reported that they recalled dreams *several times a week* or *almost every morning* over the past few months. In addition, 52.00% of the preteens reported that they experienced nightmares, and 45.48% lucid dreams, *less than once a month* or *never* over the past few months. No gender differences were observed in dream variables. Nocturnal awakenings were linked to all dream variables, while sleep duration and sleep efficiency were related to nightmare frequency and emotions in dreams. Importantly, sleep duration and sleep efficiency were not associated with dream recall nor lucid dream frequency, with Bayesian analyses supporting the null hypothesis. These findings offer a comprehensive understanding of preteens’ dreams and their connection to key sleep aspects.

## 1 INTRODUCTION

Dreaming is a subjective experience that occurs during sleep, and refers to the production of mental representations by the sleeping brain (Schredl, 2010). Upon awakening, one may eventually remember, and describe, all or part of these mental experiences. The frequency of the occurrence of dreams, as well as their content, fluctuates both across participants and among participants over time. Although these dream properties have been largely explored for adults (Blagrove & Pace‐Schott, 2010; Eichenlaub et al., 2017; Nir & Tononi, 2010; Schredl, 2010), large‐scale studies in childhood and adolescence are, in comparison, relatively rare. This lack of information is all the more unfortunate given that dreaming might be a proxy for neuronal development (Sándor et al., 2014).

Nightmares in children and adolescents have been the subject of several large cohort studies (e.g., Nielsen et al., 2000; Schredl et al., 2009a; Simard et al., 2008). Beyond quantifying the prevalence of nightmares in these populations (Schredl et al., 2009b), these studies found that girls tended to report more nightmares than boys, and that nightmare frequency was associated with psychopathological symptoms such as anxiety or emotional symptoms (Nielsen et al., 2000; Schredl et al., 2009a; Simard et al., 2008). In a large‐scale study involving more than 3500 children aged 6–18, the UK library study simultaneously assessed several dream properties, including dream recall frequency, nightmare frequency, and lucid dream frequency (Georgi et al., 2012; Schredl et al., 2012). The authors showed that, on average, children experienced dreams between once a month and 3 times a week, while they experienced nightmares between once a year and 4 times a month, and that these dream measures were correlated with each other (Georgi et al., 2012). In addition, 43.5% of the children reported having had at least one lucid dream in their life, and experiencing lucid dreams was related to both dream recall and nightmare frequency (Schredl et al., 2012). The relative high incidence of lucid dreams in children in comparison with adults, and the correlation with dream recall frequency has been found in another large‐scale survey (*n* = 694, aged 6–19, Voss et al., 2012). Interestingly, Georgi et al. (2012) reported small, but significant, gender effects in the dream variables mentioned above, with girls exhibiting higher dream recall frequency and nightmare frequency than boys; this gender difference being consistent with previous meta‐analyses (Schredl & Reinhard, 2008; Schredl & Reinhard, 2011). The UK library study also explored various aspects of dream content, including the emotions experienced in dreams (Schredl et al., 2019). Based on the rating of an external judge on the most recent dream the children remembered, the authors found that the intensity of negative emotions was higher than the intensity of positive emotions, and that girls experienced more intense positive emotions than boys (Schredl et al., 2019). Overall, these large‐scale studies provide important insights into the properties of children's and adolescents’ dreams.

The link between dreaming and sleep is a subject of intense research, and the identification of sleep parameters (including neural features of the sleeping brain) that may predict, or be related to, dream recall or content is of particular importance (for recent reviews, see Ruby, 2020; Scarpelli et al., 2022; Scarpelli et al., 2021). Unsurprisingly, several studies have underlined the significant impact of nightmares on sleep parameters. It has been shown that individuals with frequent nightmares report poorer sleep quality (Lancee et al., 2010; Paul et al., 2015). Accordingly, objective measurements of altered sleep architecture, including reduced sleep efficiency and increased nocturnal awakenings, have been reported in nightmare sufferers in sleep laboratory settings (Germain & Nielsen, 2003; Simor et al., 2012), while this effect was not replicated in ambulatory polysomnography settings (Paul et al., 2015). If nightmares have an impact on sleep, it is also assumed that certain sleep parameters facilitate non‐nightmarish dreams. In support of the widely accepted arousal‐retrieval model (Koulack & Goodenough, 1976), which emphasises the need for a wakefulness period to occur after a dream so that its content can be transferred from short‐term to long‐term memory and thus be retrieved, several studies have reported a relationship between dream recall frequency and nocturnal awakenings (Eichenlaub et al., 2014; Schredl et al., 2003; Vallat et al., 2017; van Wyk et al., 2019). Using retrospective questionnaires, Schredl et al. (2003) found a modest, but significant, positive correlation between dream recall frequency and the frequency of nocturnal awakenings. Similarly, by comparing the sleep structure of high‐ vs. low‐dream recallers in a full‐night polysomnography study, both the total duration of nocturnal awakenings (Eichenlaub et al., 2014; van Wyk et al., 2019) and the number of long (i.e., lasting more than 1 minute) awakenings (Vallat et al., 2017; van Wyk et al., 2019) were higher in high‐dream recallers. Importantly, no other sleep variables exhibited a difference between high‐ vs. low‐dream recallers, despite an extensive analysis of both the macro‐ and microstructure of sleep (Vallat et al., 2017; van Wyk et al., 2019). Taken together, these findings highlight the interest in considering sleep variables, and in particular measures of sleep quality and nocturnal awakenings, in relation to dream properties, but also raise the question of whether the effects described in adults can be replicated in the younger population.

The purpose of the present study was twofold. First, we sought to systematically collect dream habits from a large cohort (*n* = 1151) of preteens in the sixth grade (mainly aged 11–12 years old). Second, we sought to study the relationship between these dream habits and three main self‐reported sleep parameters i.e., sleep duration, sleep efficiency, and nocturnal awakenings.

## 2 METHODS

The study was part of a larger project assessing the relationship between physical activity and attention in middle schools (Pinelli et al., 2023). Part of the data were presented in a previous study that explored the relationship between sleep habits, attention, and class climate (Eichenlaub et al., 2023).

### 2.1 Participants

Data were collected between June 2021 and March 2022 in 1151 preteens enrolled in the sixth grade (597 boys and 554 girls; mean age ± SD; 11.31 ± 0.62 years old; 96.00% aged 11–12). There was no age difference between the girls and boys (*t*(1141) = −0.154, *p* = 0.877). This study was conducted in compliance with the Declaration of Helsinki and was approved by the ethics committee of CER Grenoble Alpes (Avis‐ 2020‐09‐01‐4).

### 2.2 Sleep measures

The sleep measures are described in detail in Eichenlaub et al. (2023), and assessed sleep habits over the past few months. Briefly, the typical bedtime (“*What time did you usually go to bed?*”) and waking time (“*What time did you usually get up?*”), the duration to fall asleep (i.e., “*How long did it take you to fall asleep?*”; i.e., sleep onset latency, SOL) and the time spent awake during the night (“*During the night, how long were you awake in total?*”; i.e., wake after sleep onset, WASO) were collected separately for weekdays and weekends. In addition, subjective sleep need (SSN) was collected, without distinction between weekdays and weekends.

During data collection, two additional measurements of nocturnal awakenings were included, separately for weekdays and weekends. The first measure assessed the number of awakenings during the night (from 0 to more than 10 times) using the question “*How many times did you wake up during the night?*” The second measure assessed the feeling of being woken up during the night regardless of the cause of the awakening (using a 5‐point scale, i.e., “never” [1], “rarely” [2], “sometimes” [3], “often” [4], “very often” [5]) with the question “*Did you feel like you were being woken up during the night?*”

Time in bed (TIB) was computed as the duration from bedtime to waking time, sleep duration as TIB minus SOL and WASO, and sleep efficiency as sleep duration divided by TIB and multiplied by 100.

As for the three measures of nocturnal awakenings, and to avoid any confusion, the duration of staying awake during the night will be referred as WASO, while the number of awakenings during the night and the overall feeling of being woken up at night will be referred to as the number of awakenings and feeling awaken, respectively.

### 2.3 Dream measures

Dream measures were assessed using a questionnaire derived from the Mannheim dream questionnaire (Schredl et al., 2014), which demonstrates high test–retest reliability (Ghorayeb et al., 2019; Scapin et al., 2018; Schredl et al., 2014). The dream recall frequency was assessed using a 7‐point scale (“never” [1], “less than once a month” [2], “about once a month” [3], “two or three times a month” [4], “about once a week” [5], “several times a week” [6], and “almost every morning” [7]). A dream was defined as “when you sleep, you may have images and stories in your mind that you remember afterward after waking up”, and the question was “*Over the past few months, how often have you remembered your dreams?*”

For eliciting nightmare and lucid dream frequencies, the same 7‐point scale as described above was applied. A nightmare was defined as a “bad dream that wakes you up and that you remember easily after waking up”, and the question was “*Over the past few months, how often have you had nightmares?*” A lucid dream was defined as a “dream in which you realise you are dreaming and in which you can, possibly, change the dream's story”, and the question was “*Over the past few months, how often have you had lucid dreams?*”

The intensity of both negative and positive emotions was assessed separately and using a 4‐point scale (“not at all negative,” “somewhat negative,” “moderately negative,” and “very negative,” and its counterpart for positive emotions). It was first stated that “when you dream, you can feel emotions. These emotions can be negative (e.g., sadness, fear, anger) or positive (e.g., joy, pride, happiness).” Following this statement, the question was “*Over the past few months, how strong were the negative emotions you felt in your dreams?*” and its counterpart for positive emotions.

### 2.4 Socio‐demographic measures

A sociodemographic questionnaire was used to collect information about age, gender, native language, and parental occupation. In addition, private/public status, urban/rural status, school size, educational priority networks status, school schedule, and number of hours of class per week were collected from teachers and principals. These sociodemographic characteristics can be found in Eichenlaub et al. (2023).

### 2.5 Procedure

The students filled out the questionnaires online (using Qualtrics) and individually in a computer room at school. The session lasted about 1 hour and was supervised by a teacher who was initially briefed by a researcher in psychology.

### 2.6 Statistical analysis

Bayesian statistics were applied for the correlation analysis and the independent samples comparison. Because of the nature of the data (i.e., ordinal scales) and the fact that the data did not follow a normal distribution, non‐parametric tests were chosen. To test the relationship among the dream variables but also between the dream variables and the main sleep parameters, Kendall's rank correlation coefficient (Kendall *t*) was applied. To test a gender effect on the dream variables, a Mann–Whitney U‐test was applied. The statistical analyses were performed using JASP (van Doorn et al., 2021; Wagenmakers, Love, et al., 2018; Wagenmakers, Marsman, et al., 2018). Statistical evidence was reported using Bayes factor (BF), and its interpretation followed the classification scheme as presented in Wagenmakers, Love, et al. (2018). The default prior parameters were used for both the correlation analysis (i.e., stretched beta prior width = 1.0) and the Mann–Whitney U‐test (i.e., Cauchy prior width = 0.707), and, when available, robustness analysis was performed to test the evidential impact of these prior settings (i.e., effect of these prior settings on the BF).

A Bayes factor between 10 and 30 was considered as “strong” (*), a BF between 30 and 100 as “very strong” (**), and a BF above 100 as “decisive/extreme” (***) evidence in favour of H_1_. On the contrary, a BF between 1/10 and 1/30 was considered as “strong” (^**#**^), a BF between 1/30 and 1/100 as “very strong” (^**##**^), and a BF below 1/100 as “decisive/extreme” (^**###**^) evidence in favour of H_0_.

## 3 RESULTS

### 3.1 Dream habits

**FIGURE 1:**
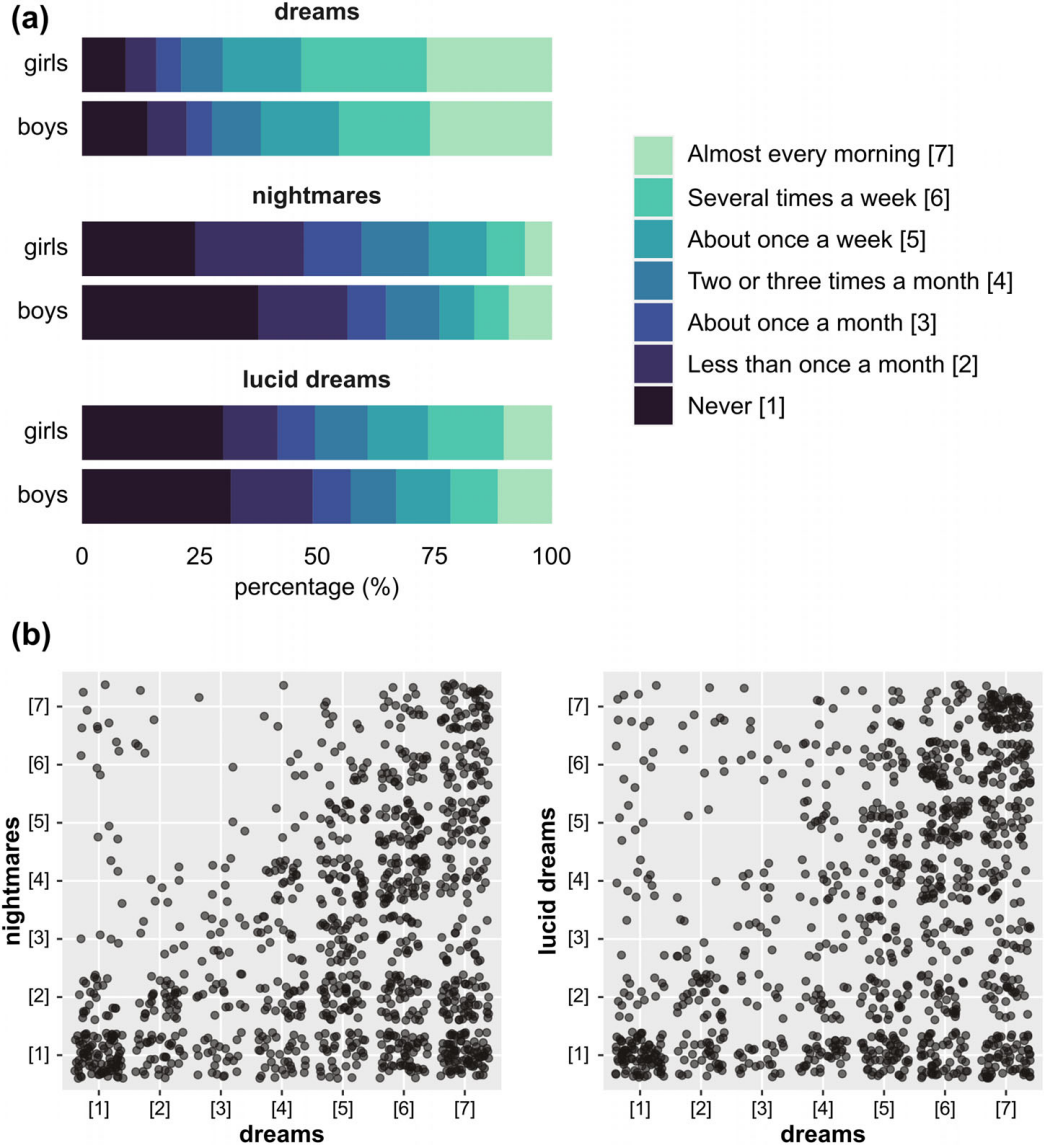
Frequency of dreams, nightmares, and lucid dreams and inter‐correlations. (a) Likert plots of the dream recall, nightmare and lucid dream frequency for the boys and the girls, separately. For each type of dream, the frequency was assessed on a 7‐point scale as follows: “never” [1], “less than once a month” [2], “about once a month” [3], “two or three times a month” [4], “about once a week” [5], “several times a week” [6], and “almost every morning” [7]. (b) Correlations (Kendall *t*) between dream recall and nightmare frequency (*t* = 0.249, BF = 1.02e+33***) and between dream recall and lucid dream frequency (*t* = 0.285, BF = 3.02e+43***). BF: Bayes factor. *** BF > 100 i.e., “decisive/extreme” evidence in favour of a correlation (in favour of H_1_)

**FIGURE 2:**
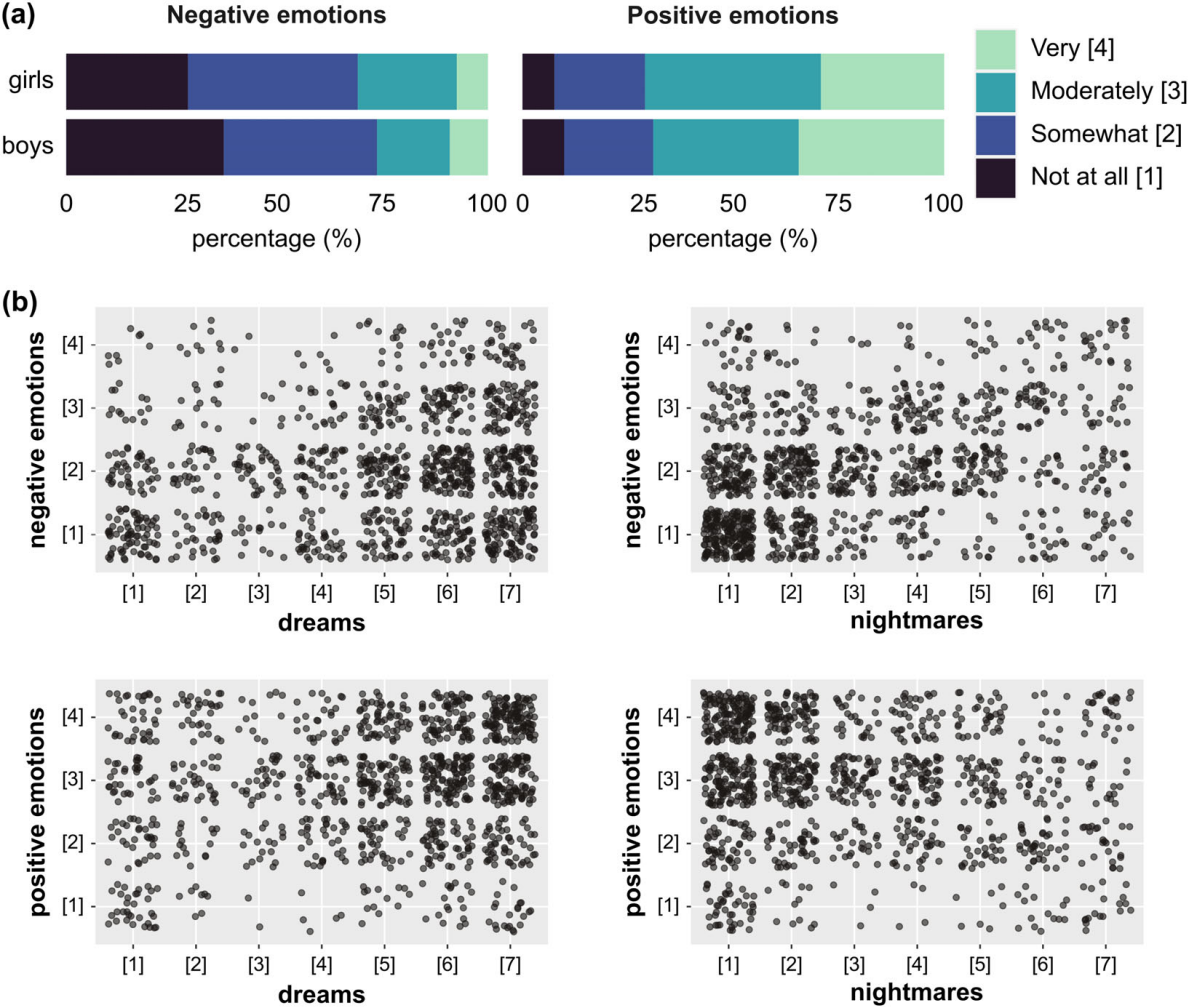
Intensity of negative and positive emotions experienced in dreams and correlations with dream recall and nightmare frequency. (a) Likert plots of the intensity of negative and positive emotions experienced in dreams and for the boys and the girls, separately. The emotional intensity was assessed on a 4‐point scale as follows: “not at all negative,” “somewhat negative,” “moderately negative,” and “very negative,” and its counterparts for positive emotions. (b) Correlations (Kendall *t*) between the intensity of negative emotions and dream recall frequency (*t* = 0.108, BF = 1.19e+5***) and nightmare frequency (*t* = 0.285, BF = 1.11e+44***), and between the intensity of positive emotions and dream recall frequency (*t* = 0.115, BF = 9.12e+5***) and nightmare frequency (*t* = −0.135, BF = 6.07e+8***). BF: Bayes factor. *** BF > 100 i.e., “decisive/extreme” evidence in favour of a correlation (in favour of H_1_)

Dream measures are summarised in Figures 1 and 2, and Tables 1 and 2. As for the dream recall frequency, the vast majority of the preteens (65.88%) reported having dreams in mind *at least about once a week*, including 49.21% with dreams in mind *several times a week* or *almost every morning*. Not surprisingly, the frequency of nightmare and lucid dream presented a reverse pattern, with 52.00% and 45.48% of the preteens reporting having nightmares or lucid dreams *less than once a month* or *never*, respectively. The dream recall frequency was positively correlated with nightmare (*t* = 0.249, BF = 1.02e+33***) and lucid dream (*t* = 0.285, BF = 3.02e+43***) frequency, and nightmare frequency was positively correlated with lucid dream frequency (*t* = 0.155, BF = 9.20e+11***), with Bayes factors indicating “decisive/extreme” evidence in favour of a correlation (Figure 1).

**TABLE 1:** Frequency of dreams, nightmares, and lucid dreams for all the children and for the boys and the girls, separately.

	Dream recall frequency			Nightmare frequency			Lucid dream frequency		
	All	Boys	Girls	All	Boys	Girls	All	Boys	Girls
Almost every morning [7]	26.32%	25.98%	26.68%	7.57%	9.21%	5.79%	10.96%	11.56%	10.31%
Several times a week [6]	22.90%	19.36%	26.68%	7.74%	7.37%	8.14%	12.96%	10.05%	16.09%
About once a week [5]	16.67%	16.64%	16.70%	9.74%	7.37%	12.30%	12.17%	11.56%	12.84%
Two or three times a month [4]	9.65%	10.36%	8.89%	12.78%	11.39%	14.29%	10.44%	9.72%	11.21%
About once a month [3]	5.35%	5.43%	5.26%	10.17%	8.21%	12.30%	8.00%	8.04%	7.96%
Less than once a month [2]	7.46%	8.32%	6.53%	20.96%	18.93%	23.15%	14.61%	17.42%	11.57%
Never [1]	11.67%	13.92%	9.26%	31.04%	37.52%	24.05%	30.87%	31.66%	30.02%

*Note*: Dream habits were estimated by the children over the past few months. The frequency of dream recall, nightmare, and lucid dream were assessed on a 7‐point scale as follows: “never” [1], “less than once a month” [2], “about once a month” [3], “two or three times a month” [4], “about once a week” [5], “several times a week” [6], and “almost every morning” [7].

**TABLE 2:** Negative and positive emotions in dreams for all the children and for the boys and the girls, separately.

	Negative emotions			Positive emotions		
	All	Boys	Girls	All	Boys	Girls
Very [4]	8.25%	9.05%	7.40%	31.97%	34.51%	29.24%
Moderately [3]	20.24%	17.25%	23.47%	37.97%	34.51%	41.70%
A little [2]	38.23%	36.35%	40.25%	21.29%	21.11%	21.48%
Not at all [1]	33.28%	37.35%	28.88%	8.78%	9.88%	7.58%

*Note*: Negative emotions in dreams were assessed on a 4‐point scale as follows: “not at all negative” [1], “somewhat negative” [2], “moderately negative” [3], and “very negative” [4], and its counterpart for positive emotions.

As for the emotions experienced in the dreams, 71.50% of the preteens reported experiencing negative emotions *a little* or *not at all*, while 69.94% reported experiencing positive emotions in dreams *moderately* or *very*. The intensity of negative emotions experienced in dreams was positively correlated with dream recall frequency (*t* = 0.108, BF = 1.19e+5***), nightmare frequency (*t* = 0.285, BF = 1.11e+44***), and lucid dream frequency (*t* = 0.089, BF = 1060***). The intensity of positive emotions experienced in dreams was positively correlated with dream recall frequency (*t* = 0.115, BF = 9.12e+5***), and lucid dream frequency (*t* = 0.082, BF = 236***), but negatively correlated with nightmare frequency (*t* = −0.135, BF = 6.07e+8***). For all these correlations, Bayes factors indicated “decisive/extreme” evidence in favour of a correlation (Figure 2).

### 3.2 Gender differences

Bayesian Mann–Whitney U tests were used to compare boys vs. girls on dream variables. The results indicated weak (or “anecdotal”) evidence in favour of either a difference (in favour of H_1_) or a non‐difference (in favour of H_0_) between boys and girls for each of the following dream variables: dream recall frequency (BF = 0.77), nightmare frequency (BF = 1.60), lucid dream frequency (BF = 0.17), and negative emotions experienced in dreams (BF = 0.47). However, for the positive emotions experienced in dreams, the results indicate “strong” evidence in favour of a non‐difference, showing that the positive emotional intensity did not differ between boys and girls (BF = 0.07^**#**^).

### 3.3 Relationship between dream and sleep variables

**FIGURE 3:**
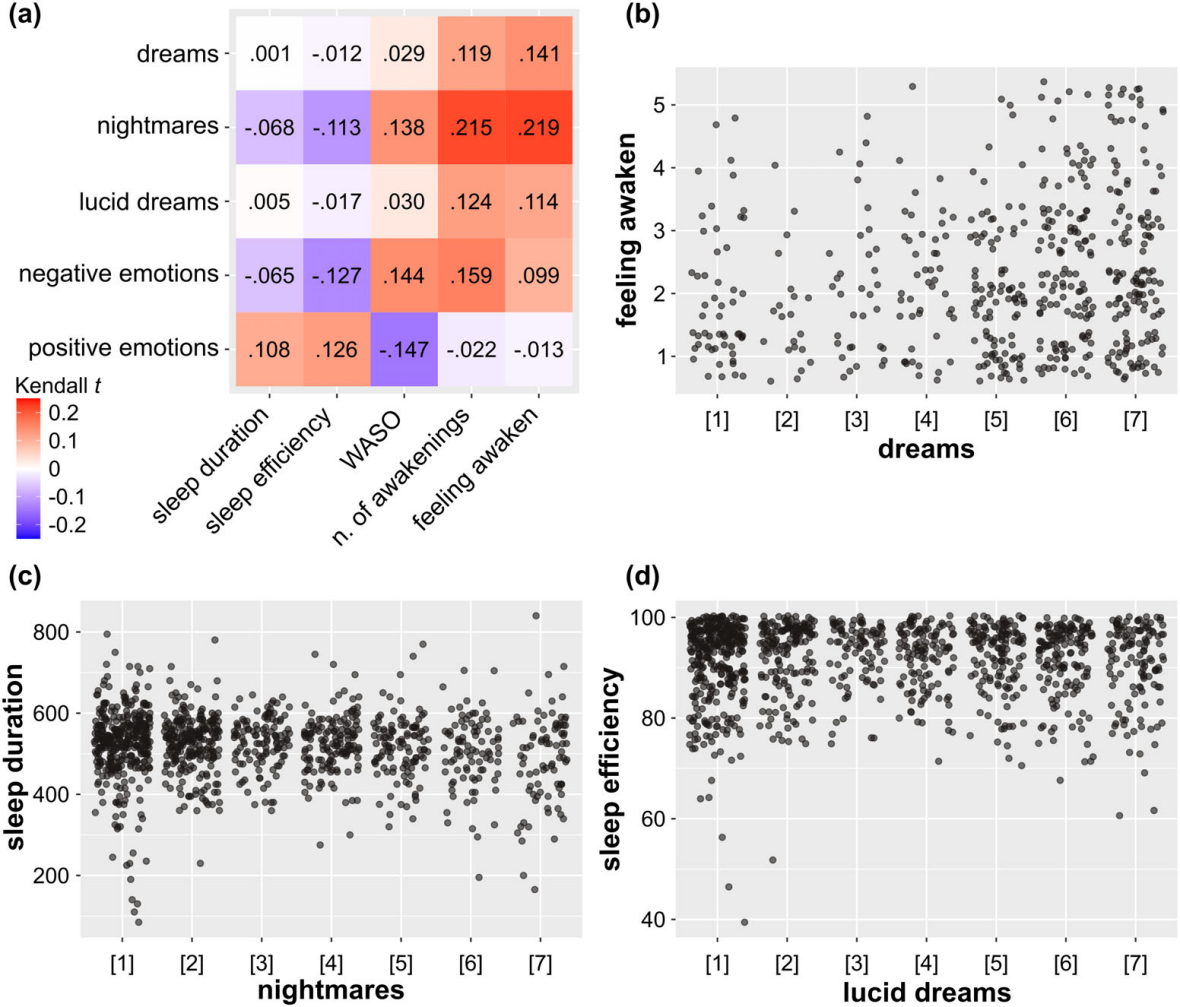
Correlations between the dream and sleep variables. (a) Heatmap of bivariate correlation (Kendall *t*) between the dream variables (i.e., dream recall, lucid dream, and nightmare frequency, and the intensity of positive and negative emotions experienced in dreams) and the sleep variables (i.e., sleep duration, sleep efficiency and the three nocturnal awakening measures). (b) Correlation (Kendall *t*) between dream recall frequency and the feeling of being woken up (*t* = 0.141, BF = 5.79e+3***). (c) Correlation (Kendall *t*) between nightmare frequency and sleep duration (*t* = −0.068, BF = 14.88*). (d) Correlation (Kendall *t*) between lucid dream frequency and sleep efficiency (*t* = −0.017, BF = 0.06^**#**^). * BF between 10 and 30 i.e., “*strong*” evidence in favour of a correlation (in favour of H_1_). *** BF > 100 i.e., “*decisive/extreme*” evidence in favour of a correlation (in favour of H_1_). ^**#**^ BF between 1/10 (0.1) and 1/30 (0.03) i.e., “*strong*” evidence in favour of a non‐correlation (in favour of H_0_)

Three main self‐reported sleep parameters, i.e., sleep duration, sleep efficiency and nocturnal awakenings, were considered. Nocturnal awakenings were assessed in terms of: (i) the duration of staying awake during the night (i.e., *WASO*); (ii) the number of awakenings during night (i.e., *number of awakenings*); and (iii) the overall feeling of being woken up at night (i.e., *feeling awake*). Only the sleep data collected for the weekdays are presented here, and the results of this analysis are displayed in Figure 3 and Table 3 (see also Supplementary Table 1).

**TABLE 3:** Correlations between the dream and sleep variables.

	Sleep duration	Sleep efficiency	WASO	Number of awakenings	Feeling awake
Dream recall frequency	*t* = 0.001	*t* = −0.012	*t* = 0.029	*t* = 0.119	*t* = 0.141
	BF = 0.04^#^	BF = 0.05^#^	BF = 0.12	BF = 189.42***	BF = 5.79e+3***
	(*n* = 1140)	(*n* = 1140)	(*n* = 1140)	(*n* = 516)	(*n* = 516)
Nightmare frequency	*t* = −0.068	*t* = −0.113	*t* = 0.138	*t* = 0.215	*t* = 0.219
	BF = 14.88*	BF = 5.78e+5***	BF = 1.92e+9***	BF = 4.07e+10***	BF = 9.32e+10***
	(*n* = 1150)	(*n* = 1150)	(*n* = 1150)	(*n* = 527)	(*n* = 527)
Lucid dream frequency	*t* = 0.005	*t* = −0.017	*t* = 0.030	*t* = 0.124	*t* = 0.114
	BF = 0.04^#^	BF = 0.06^#^	BF = 0.12	BF = 454.72***	BF = 126.30***
	(*n* = 1150)	(*n* = 1150)	(*n* = 1150)	(*n* = 527)	(*n* = 527)
Negative emotions	*t* = −0.065	*t* = −0.127	*t* = 0.144	*t* = 0.159	*t* = 0.099
	BF = 8.54	BF = 4.79e+7***	BF = 1.42e+10***	BF = 1.56e+5***	BF = 17.25*
	(*n* = 1151)	(*n* = 1151)	(*n* = 1151)	(*n* = 527)	(*n* = 527)
Positive emotions	*t* = 0.108	*t* = 0.126	*t* = −0.147	*t* = −0.022	*t* = −0.013
	BF = 1.27e+5***	BF = 3.32e+7***	BF = 5.35e+10***	BF = 0.08^#^	BF = 0.06^#^
	(*n* = 1151)	(*n* = 1151)	(*n* = 1151)	(*n* = 527)	(*n* = 527)

*Note*: Kendall *t*, Bayes factor (BF) and sample size (n) of the correlations between the dream variables and the sleep variables.

^#^ BF between 1/10 and 1/30 i.e., “*strong*” evidence in favour of a non‐correlation (in favour of H_0_).

* BF between 10 and 30 i.e., “*strong*” evidence in favour of a correlation (in favour of H_1_).

*** BF > 100 i.e., “*decisive/extreme*” evidence in favour of a correlation (in favour of H_1_).

Dream recall frequency and lucid dream frequency were positively correlated with the number of awakenings during the night (dreams: *t* = 0.119, BF = 189.42***; lucid dreams: *t* = 0.124, BF = 454.72***), and the feeling of being woken up at night (dreams: *t* = 0.141, BF = 5.79e+3***; lucid dreams: *t* = 0.114, BF = 126.30***), with Bayes factors (BF) exhibiting “decisive/extreme” evidence in favour of a correlation. On the contrary, dream recall frequency and lucid dream frequency did not correlate with sleep duration (dreams: *t* = 0.001, BF = 0.04^**#**^; lucid dreams: *t* = 0.005, BF = 0.04^**#**^) and sleep efficiency (dreams: *t* = −0.012, BF = 0.05^**#**^; lucid dreams: *t* = −0.017, BF = 0.06^**#**^), with Bayes factors exhibiting “strong” evidence in favour of a non‐correlation (in favour of H_0_).

Nightmare frequency was positively correlated with all nocturnal awakening measurements (WASO: *t* = 0.138, BF = 1.92e+9***; number of awakenings: *t* = 0.215, BF = 4.07e+10***; feeling awake: *t* = 0.219, BF = 9.32e+10***), and negatively correlated with sleep duration (*t* = −0.068, BF = 14.88*) and sleep efficiency (*t* = −0.113, BF = 5.78e+5***) with Bayes factors (BF) exhibiting “strong” or “decisive/extreme” evidence in favour of a correlation.

The intensity of negative emotions experienced in dreams was negatively correlated with sleep efficiency (*t* = −0.127, BF = 4.79e+7***) and positively correlated with nocturnal awakenings (WASO: *t* = 0.144, BF = 1.42e+10***; number of awakenings: *t* = 0.159, BF = 1.56e+5***; feeling awake: *t* = 0.099, BF = 17.25*) with Bayes factors exhibiting “strong” or “decisive/extreme” evidence in favour of a correlation.

Inversely, the intensity of positive emotions experienced in dreams was positively correlated with sleep duration (*t* = 0.108, BF = 1.27e+5***) and sleep efficiency (*t* = 0.126, BF = 3.32e+7***), and negatively correlated with WASO (*t* = −0.147, BF = 5.35e+10***) with Bayes factors (BF) exhibiting “decisive/extreme” evidence in favour of a correlation. In addition, the analysis also showed that positive emotions in dreams did not correlate with both the number of awakenings (*t* = −0.022, BF = 0.08^**#**^) and the feeling of being woken up at night (*t* = −0.013, BF = 0.06^**#**^) with Bayes factors exhibiting “strong” evidence in favour of a non‐correlation (in favour of H_0_).

## 4 DISCUSSION

### 4.1 Dream habits in preteens

In the present study, the vast majority of the preteens (65.88%) reported having dreams in mind at least *about once a week*, with almost half of them (49.21%) having dreams in mind *several times a week* or *almost every morning*. Overall, these patterns are in accordance with previous studies using the same 7‐point scale questionnaire in adults (Napias et al., 2021; Scapin et al., 2018; Schredl et al., 2014; Schredl & Göritz, 2017). However, it is important to note substantial variability between studies (see Supplementary Figure 1). For instance, the proportion of adult participants experiencing dreams *several times a week* or *almost every morning* varies from 13.20% to 57.17% (Napias et al., 2021; Scapin et al., 2018; Schredl et al., 2014; Schredl & Göritz, 2017). Beyond possible selection bias in recruiting participants across studies, part of this variation can be explained by the relationship between dream recall frequency and age, as the well‐known progressive decrease in dream recall frequency with advancing age in adults is preceded by an increase during adolescence and early adulthood (Nielsen, 2012).

As for the emotions experienced in dreams, we found that the preteens reported more intense positive than negative emotions, in apparent contradiction with previous results in both adults and children (Schredl et al., 2019; Schredl & Doll, 1998) and the so‐called *negativity bias* in dreams (e.g., Valli et al., 2008). However, as pointed out in several previous studies, this predominance of negative emotions was observed only when external judges rated the emotions in dreams, whereas, in the case of self‐ratings (i.e., as in the present study), the ratio was more balanced (Conte et al., 2020; Röver & Schredl, 2017; Schredl & Doll, 1998), or even in favour of positively valenced dreams (Sikka et al., 2014). This *positivity bias* in emotions experienced in dreams, confirmed in preteens in the present study, needs to be explored further and in light of the putative role of dreaming in emotional processes (Scarpelli et al., 2019).

### 4.2 Dream variables are related to each other

Not surprisingly, the different dream variables assessed in the present study were all related to each other. Dream recall, nightmare, and lucid dream frequency were positively correlated with each other, but also positively correlated with the intensity of negative emotions in dreams. Finally, while dream recall frequency and lucid dream frequency were also positively correlated with the intensity of positive emotions in dreams, nightmare frequency was negatively correlated with the latter. These inter‐correlations across dream variables, as well as their direction, are largely in accordance with the literature (Georgi et al., 2012; Schonbar, 1961; Schredl, 2003; Schredl et al., 2012; Schredl & Doll, 1998; Vallat et al., 2018), and suggest potential mechanisms at play in dreaming. For instance, the relationship between the emotional intensity experienced in dreams, irrespective of its valence, and the dream recall frequency might be explained by the salience hypothesis (Cohen & MacNeilage, 1974), according to which emotions experienced during dreams facilitate the subsequent recall of the latter. Although initially formulated for dreams, this hypothesis seems plausible for nightmares as well, as the latter are particularly negative, but also for lucid dreams, as greater self‐awareness might also facilitate later recall of the experience.

### 4.3 Inconclusive evidence for a gender effect in dreaming

In the present sample of preteens (96.00% of them being aged 11–12 years old), we found inconclusive evidence for or against a gender effect in all the dream variables assessed, except for the intensity of positive emotions. In fact, Bayesian analyses revealed “strong” evidence in favour of the null hypothesis, that is, boys and girls do not differ in the intensity of positive emotions experienced in their dreams.

As for dream recall and nightmare frequency, our results are in apparent contradiction with the literature (Georgi et al., 2012; Schredl et al., 2012; Schredl & Reinhard, 2008; Schredl & Reinhard, 2011). However, while compiling evidence suggest a gender effect in adults, meta‐analyses reported that in children aged 10 or under, the gender difference for nightmare frequency was not significant (Schredl & Reinhard, 2011), while, for dream recall frequency, the effect size was the smallest compared with older age groups (Schredl & Reinhard, 2008). In addition, given the substantial brain changes that the children undergo during these years (Casey et al., 2008), the use of broad age ranges (e.g., children aged 10–18 grouped within a single age group), as usually seen in the literature, might have introduced a bias, with a gender effect largely driven by older adolescents. This potential limitation can be also applied in the very few studies having reported a gender effect in lucid dream frequency (Schredl et al., 2012). Together, our results stress the need of future studies using a narrow age‐range when exploring a gender effect in dream variables in children, and suggest that gender differences in dreaming might gradually emerge during adolescence, possibly triggered, at least to some extent, by the emergence of sexual dimorphism in the adolescent brain (Giedd et al., 1997; Lenroot & Giedd, 2010).

### 4.4 The role of nocturnal awakenings in dreams recall

In accordance with studies in adults (De Gennaro et al., 2010; Eichenlaub et al., 2014; Schredl et al., 2003; Vallat et al., 2017; van Wyk et al., 2019), we replicated, in preteens, a relationship between dream recall frequency and nocturnal awakenings. Our results provide further support for the arousal–retrieval model (Koulack & Goodenough, 1976), which emphasises the need for a period of wakefulness following a dream so that its content can be transferred from short‐term to long‐term memory and thus be retrieved (for a recent review, see also Nemeth, 2023). Interestingly, we found a relationship when the preteens were asked to assess the number of awakenings or the feeling of being awakened during the night, but not when they were assessing the total duration of being awake at night. This finding may be explained by the difficulty for a sleeper to accurately estimate nocturnal awakenings (Åkerstedt et al., 1994; Baker et al., 1999; Mazza et al., 2020; Tremaine et al., 2010), the total time spent awake during the night being particularly difficult to assess. Furthermore, this time spent awake during the night does not necessarily depend on the number of awakenings, and even short awakenings of approximately 2 minutes may be sufficient for a successful dream encoding and subsequent recall (Vallat et al., 2017; van Wyk et al., 2019), highlighting the interest in considering both duration and number of awakenings.

### 4.5 Nightmare frequency, but not lucid dream frequency, is related to sleep quality

In the present study, nightmare frequency was negatively related to both sleep duration and sleep efficiency while positively related to nocturnal awakenings. These correlations corroborate the potentially harmful impact of nightmares on sleep (Germain & Nielsen, 2003; Lancee et al., 2010; Paul et al., 2015; Simor et al., 2012). In addition, a variety of mental disorders, including mood and anxiety disorders, have been associated with nightmare frequency (Levin & Fireman, 2002; Li et al., 2010; Rek et al., 2017; Sandman et al., 2015); these disorders being also related to insufficient or disturbed sleep (for recent reviews, see Chellappa & Aeschbach, 2022; Palagini et al., 2022). In accordance with these results, Nielsen and Levin (2007) have proposed a neurocognitive model of nightmare aetiology, which emphasises that a certain personality trait (i.e., *affect distress*) facilitates the development and expression of nightmares.

Importantly, such an association was not found for lucid dreams. Lucid dreams refer to a particular type of dream, in which the dreamer becomes aware that he/she is dreaming, and can eventually modify the content of the dream (LaBerge, 1990). At the neural level, lucid dreaming is linked to a specific brain pattern in rapid eye movement (REM) sleep, characterised by increased activity in different regions of the frontal, parietal, and temporal cortex (Baird et al., 2019). Given the importance of sleep in various physiological and cognitive functions, this hybrid state in lucid dreaming might be viewed as potentially detrimental (Vallat & Ruby, 2019). However, the few studies that have explored the relationship between lucid dreams and sleep quality report mixed results. First, studies that did find a significant relationship reported a weak association (Aviram & Soffer‐Dudek, 2018), or a relationship that disappeared when nightmare frequency was statistically controlled, suggesting that poorer sleep in lucid dreamers might be explained by their tendency to experience nightmares more often (Schadow et al., 2018). Second, in a large scale study including almost 2000 participants, Denis and Poerio (2017) did not find a significant correlation between lucid dream frequency and sleep quality, this lack of significant correlation having also been reported in a general population sample (Ribeiro et al., 2020) and by using a longitudinal (within‐subject) approach (Schredl et al., 2020). In accordance with these recent studies, we did not find such a significant correlation in the present study. In fact, Bayesian analyses revealed “strong” evidence in favour of the null hypothesis, that is, there is no correlation between lucid dream frequency and both the sleep duration and the sleep efficiency in preteens (see Table 3). Of note, it is important to mention that the aforementioned studies (including the present one) assessed sleep quality using questionnaires, without objective measures of sleep, and future polysomnography studies are needed to clarify the relationship between lucid dreams and the many facets of sleep.

### 4.6 Strengths and limitations

In dream research, a selection bias is often introduced that may affect the results, as these studies are advertised as a study focussing on dreaming, it is likely that individuals with an interest in dreaming will be more likely to take part in the experiment. The current study has no such limitations, as the dream and sleep questionnaires were systematically completed by all the children of a given class. In addition, while not designed as an epidemiological study, the data from the current study were collected from 21 French middle schools that cover various economic and social levels, and that are located in both rural and urban areas (Eichenlaub et al., 2023).

In the current study, self‐report questionnaires were used to assess sleep, raising the question of the accuracy of this type of measurement. Of note, studies in children comparing subjective sleep measures with actigraphy reported a moderate to high correlation in various sleep variables (Mazza et al., 2020; Tremaine et al., 2010), highlighting the validity of such subjective measurements (also discussed in Eichenlaub et al., 2023). However, this type of measurement does not allow us to extract fine‐grained information about sleep, especially about its architecture and its various microarchitectural aspects.

## 5 CONCLUSION

In conclusion, the present study provides insight into the properties of dreaming and their interconnections with sleep habits in preteens aged 11–12 years old. About half of the preteens reported having dreams in mind *several times a week* or *almost every morning*, and about half of the preteens reported having nightmares and lucid dreams, less than *once a month* or *never*. As for the emotional content of dreams, the observed *positivity bias* challenges established notions of *negativity bias* in dreams. Despite prior research indicating gender differences, our study reported inconclusive evidence, emphasising the importance of considering narrow age ranges when exploring gender effect in dreaming. Finally, our study highlights the importance of nocturnal awakenings in dreams recall, confirms that nightmares are related to poorer sleep quality, but also provides evidence in favour of a non‐relationship between lucid dreams and sleep quality. Although limited by self‐report measures, our findings offer valuable contributions to our understanding of preteens’ dreaming, setting the stage for further comprehensive research in this area.

## CONFLICT OF INTEREST STATEMENT

The authors declare that they have no known competing financial interests or personal relationships that could have appeared to influence the work reported in this paper.

## Supporting information


**DATA S1** Supporting Information.

## Data Availability

All data and analysis scripts that support the findings of this study are available in a public repository (OSF: https://osf.io/x4ngq/).
