# Supplementary material for: Dream habits in a large cohort of preteens and their relation to sleep and nocturnal awakenings
Source: J Sleep Res. 2024 Sep 11;34(2):e14339. doi: 10.1111/jsr.14339 (PMC11911058; doi:10.1111/jsr.14339)
Supplement: Supplementary file 1 — DATA S1 Supporting Information. [file JSR-34-e14339-s001.docx]

**Supplemental Information**

**Dream habits in a large cohort of preteens and their relation to sleep and nocturnal awakenings**

Jean-Baptiste Eichenlaub^1,2,*^, Romain Bouet^3^, Mathieu Pinelli^4^ and Sophie Portrat^1^

^1^ Univ. Grenoble Alpes, Univ. Savoie Mont Blanc, CNRS, LPNC, 38000 Grenoble, France

^2^ Institut Universitaire de France (IUF)

^3^ Lyon Neuroscience Research Center, INSERM U1028 / CNRS UMR 5292, Lyon, France

^4^ Univ. Grenoble Alpes, LaRAC, 38000 Grenoble, France

***** Department of Psychology, Université Savoie Mont Blanc, Domaine univ. de Jacob Bellecombette, Bât. 6/7, 73011 Chambéry, Email address: [jean-baptiste.eichenlaub@univ-smb.fr](mailto:jean-baptiste.eichenlaub@univ-smb.fr)

#

|  | **Sleep duration** | **Sleep efficiency** | **WASO (cumulative awakening)** | **Number of awakenings** |
| --- | --- | --- | --- | --- |
| **Sleep efficiency** | *t* = .465  BF = 3.33e+119***  (n = 1151) |  |  |  |
| **WASO (cumulative awakening)** | *t* = -.354  BF = 6.80e+68***  (n = 1151) | *t* = -.627  BF = 2.52e+218***  (n = 1151) |  |  |
| **Number of awakenings** | *t* = -.179  BF = 9.42e+6***  (n = 527) | *t* = -.247,  BF = 2.18e+14***  (n = 527) | *t* = .352,  BF = 2.09e+30***  (n = 527) |  |
| **Feeling awaken** | *t* = -.188,  BF = 6.64e+7***  (n = 527) | *t* = -.250,  BF = 5.90e+14***  (n = 527) | *t* = .287,  BF = 5.74e+19***  (n = 527) | *t* = .466  BF = 1.27e+54***  (n = 527) |

**Supplementary Table 1. Correlations across the sleep variables.** Kendall *t*, Bayes Factors (BF) and sample size (n) of the correlations between sleep variables. *** BF > 100 i.e., “*decisive/extreme*” evidence in favor of a correlation (in favor of H_1_).


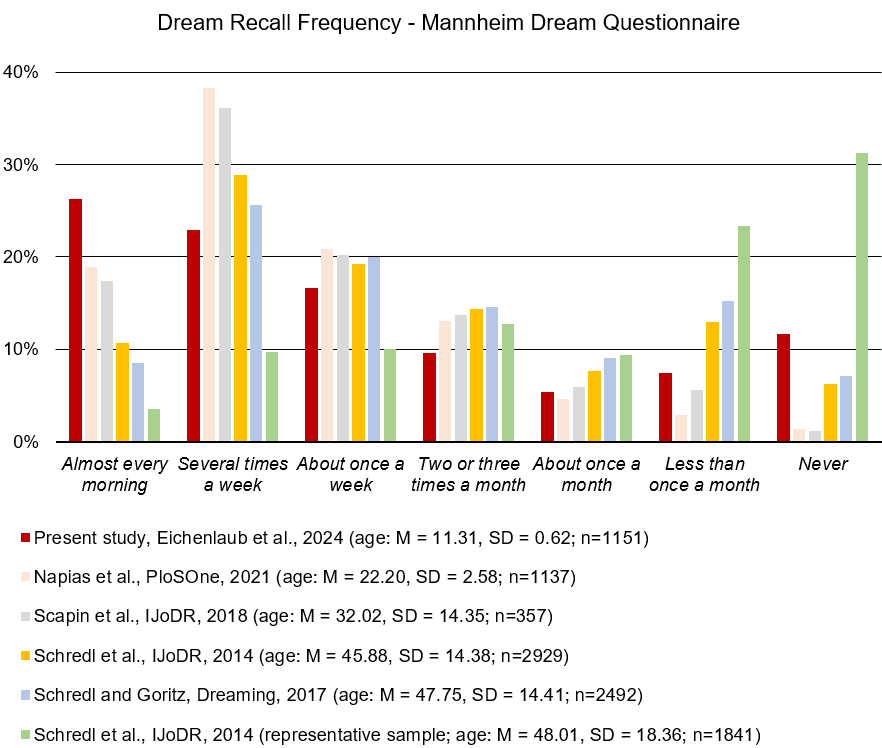


**Supplementary Figure 1.** Comparaison of the dream recall frequency assessed using the Mannheim Dream Questionnaire. For each study, the age (Mean, SD) and the total number of participants are provided [(Napias et al., 2021; Scapin et al., 2018; Schredl et al., 2014; Schredl and Göritz, 2017)](https://paperpile.com/c/yODZtg/vCu4+9DYb+LEzl+4RFd).

**References**

[Napias, A., Denechere, E., Mayo, W., Ghorayeb, I. Assessment of dream-related aspects and beliefs in a large cohort of French students using a validated French version of the Mannheim Dream questionnaire. PLoS One, 2021, 16: e0247506.](http://paperpile.com/b/yODZtg/vCu4)

[Scapin, F., Dehon, H., Englebert, J. Assessment of a French version of the Mannheim Dream questionnaire (MADRE) in a Belgian sample. International Journal of Dream Research, 2018, 11: 46–53.](http://paperpile.com/b/yODZtg/9DYb)

[Schredl, M., Berres, S., Klingauf, A., Schellhaas, S., Göritz, A.S. The Mannheim Dream questionnaire (MADRE): Retest reliability, age and gender effects. International Journal of Dream Research, 2014, 141–147.](http://paperpile.com/b/yODZtg/LEzl)

[Schredl, M., Göritz, A.S. Dream recall frequency, attitude toward dreams, and the Big Five personality factors. Dreaming, 2017, 27: 49–58.](http://paperpile.com/b/yODZtg/4RFd)
